# In Silico Assessment of Limited Blood Sampling Strategies for Individualised Pharmacokinetic‐guided Dosing of Efanesoctocog Alfa in Haemophilia A Patients

**DOI:** 10.1111/hae.70169

**Published:** 2025-11-25

**Authors:** Jelien den Hollander, Marjon H. Cnossen, Ron A. A. Mathôt

**Affiliations:** ^1^ Hospital Pharmacy‐Clinical Pharmacology, Academic Medical Centre Amsterdam Amsterdam the Netherlands; ^2^ Department of Pediatric Hematology and Oncology, Erasmus MC Sophia Children's Hospital University Medical Centre Rotterdam Rotterdam the Netherlands

**Keywords:** efanesoctocog alfa, factor VIII, haemophilia A, limited sampling strategy, pharmacokinetics

## Abstract

**Introduction:**

Efanesoctocog alfa is a novel factor VIII (FVIII) concentrate with a unique molecular design that enables Von Willebrand Factor‐independent clearance in patients with haemophilia A. Limited sampling strategies (LSSs) are necessary to implement accurate pharmacokinetic (PK)‐guided dosing for efanesoctocog alfa in clinical practice.

**Aim:**

This in silico study aims to evaluate the predictive performance of 10 LSSs with one to three samples for estimating individual PK profiles of efanesoctocog alfa.

**Methods:**

Monte Carlo simulations based on a published population PK model generated individual FVIII activity‐time profiles for a virtual population. LSSs were applied to sample from these profiles, and PK parameters, FVIII activity peak level at 0.5 h (C_0.5_), FVIII activity trough level at 168 h (C_168_) and time above FVIII activity thresholds (5, 10 and 40 IU/dL) were estimated using Bayesian forecasting.

**Results:**

All LSSs complied with our requirements of a relative mean prediction error of <±5% and a relative root mean square error of <25%. For prophylactic dosing advice, a LSS was considered clinically suitable if at least 80% of predicted C_168_ had a prediction error within −3 to 3 IU/dL. This criterion was met by one two‐sample LSS and three three‐sample LSSs. For pre‐operative dosing advice, suitability required at least 80% of predicted C_0.5_ within –20 to 20 IU/dL, fulfilled by one two‐sample and six three‐sample LSSs.

**Conclusions:**

Several LSSs demonstrated adequate predictive performance for PK‐guided dosing of efanesoctocog alfa.

## 1 Introduction

Efanesoctocog alfa is a novel recombinant factor VIII (FVIII) concentrate approved for prophylactic, perioperative and on‐demand treatment in patients with haemophilia A [1]. It features a unique molecular design that includes an Fc domain, two XTEN polypeptides and a Von Willebrand Factor (VWF) D'D3 FVIII binding domain, which prevents endogenous VWF from binding to the recombinant FVIII [2]. This structure of efanesoctocog alfa circumvents the VWF‐imposed limit on FVIII half‐life, enabling it to achieve a mean half‐life of 47 h in patients aged >12 years [2] and 40 h in patients aged <12 years [3].

This extended half‐life allows for less frequent dosing in all treatment settings [1]. In the perioperative setting, a single pre‐operative dose of 50 IU/kg is recommended, with additional doses of 30 or 50 IU/kg every 2–3 days administered as clinically indicated [1]. In the prophylactic setting, once‐weekly dosing of 50 IU/kg is recommended across all age groups [1]. This regimen maintains FVIII activity levels >40 IU/dL for approximately 4 days in patients aged >12 years [2] and 3 days in patients aged <12 years [3], approaching the normal physiological FVIII activity range of 50–150 IU/dL. At the end of a dosing interval, FVIII activity levels remain above the World Federation of Haemophilia (WFH)‐recommended prophylactic target trough level of 3–5 IU/dL [2, 3, 4]. The XTEND‐1 trial showed a mean trough level of 15 IU/dL in patients aged >12 years [2], while the XTEND‐Kids study reported mean trough levels of 6 IU/dL in children under 6 years and 7 IU/dL in those aged 6–12 years [3].

This VWF‐independent clearance mechanism not only prolongs FVIII activity but also reduces interindividual pharmacokinetic (PK) variability, which in conventional FVIII concentrates is caused by differences in endogenous VWF levels and its acute‐phase responses [2]. Nonetheless, PK variability is still observed with efanesoctocog alfa [5], highlighting the potential for further treatment optimisation through PK‐guided dosing.

A PK‐guided dosing approach allows for improved haemostatic control, offering potentially more effective and cost‐efficient treatment compared to conventional bodyweight‐based dosing strategies [6, 7, 8]. A key prerequisite for implementing PK‐guided dosing is a robust population PK (PopPK) model that accurately captures interindividual variability in PK parameters [9]. Wong et al. developed a one‐compartment PopPK model with linear clearance (CL) for efanesoctocog alfa, incorporating interindividual variability in CL and volume of distribution (V), based on data from 199 adults and adolescents and 61 children across five clinical trials [10]. This PopPK model provides prior knowledge of efanesoctocog alfa's population PK parameters. In Bayesian forecasting, the model's population data and patient‐specific blood samples are combined to estimate individual PK parameters, allowing for accurate dosing advice based on limited blood samples [11]. As the timing of blood sampling within a dosing interval affects the accuracy of the individual PK parameter estimation, finding optimal blood sampling times is crucial for the reliability of each PK‐guided dosing advice. In this in silico study, we developed and assessed ten limited sampling strategies (LSSs) with one to three blood samples for efanesoctocog alfa in a virtual population to enable accurate and precise PK‐guided dosing in a prophylactic, pre‐operative and some on‐demand settings.

## 2 Methods

### 2.1 Virtual Population

A virtual population was created consisting of 10,000 adults and 2500 children. These virtual individuals differed in bodyweight and genetic background. Bodyweight acted as a covariate on both CL and V, and individuals of Asian descent were found to have a 10.4% lower CL in the PopPK model [10].

The adult virtual population was generated using the online Copula Covariate Simulator tool of Guo et al. [12] found at https://cocosim.lacdr.leidenuniv.nl. This tool is the web application of the developed copula, which is a multivariate distribution function that captures dependency structures between 12 patient‐associated covariates based on the National Health and Nutrition Examination Survey (NHANES). The used settings were: males as haemophilia A is an X‐linked disorder predominantly affecting males, an age range of 18–70 years, with bodyweight as the covariate of interest, a body mass index range of 18.5–30 kg/m^2^, all genetic backgrounds, and an unweighted copula. The unweighted copula was used because it better reflects the oversampled Asian population in the NHANES, which aligns more closely with the ethnic composition of the population used to develop the PopPK model of efanesoctocog alfa.

Since the Copula Covariate Simulator tool was not designed for children, paediatric bodyweights were instead sampled from a truncated normal distribution (mean: 35 kg, SD: 15 kg, range: 3–60 kg) in R v4.3.1 to ensure coverage beyond the adult virtual population. A genetic background was randomly assigned to each child based on the ratio of Asian and non‐Asian descendants in the adult virtual population.

### 2.2 Development of LSSs

One‐, two‐ and three‐sample LSSs were designed to minimise patient burden by reducing the number of blood samples and hospital visits (Table 1). For example, a FVIII activity peak and trough sample can be collected within a single hospital visit. All sampling times included a time window. The trough level window was narrowed to 166–168 h, reflecting clinical practice as a dose is generally given immediately after the trough level is drawn, and the peak level is usually sampled 15–30 min after dose administration, while other windows were set at every 24 h (Day ±2 h) for flexibility.

**TABLE 1:** Evaluated sampling strategies for efanesoctocog alfa.

Sampling strategy	15–30 min	22–26 h	46–50 h	70–74 h	94–98 h	118–122 h	142–146 h	166–168 h
*7 samples*								
RSS	x		x	x	x	x	x	x
*1 sample*								
LSS1								x
LSS2						x		
*2 samples*								
LSS3						x		x
LSS4	x							x
*3 samples*								
LSS5	x	x						x
LSS6	x		x					x
LSS7	x			x				x
LSS8	x				x			x
LSS9	x					x		x
LSS10	x						x	x

Abbreviations: LSS, limited sampling strategy; RSS, reference sampling strategy.

For the one‐sample LSSs, only FVIII activity samples taken during the elimination phase were tested, such as those drawn at 166–168 h (LSS1) or 118–122 h (LSS2), as these provide better predictions of the FVIII activity trough level at 168 h (C_168_) used for prophylactic PK‐guided dosing. For the two‐sample LSSs, we evaluated whether two samples drawn during the elimination phase, between 118–122 and 166–168 h (LSS3), outperformed taking a FVIII activity peak and a trough level at 0.25–0.5 and 166–168 h (LSS4). To assess the added value of a third sample and determine its optimal timing, LSS5 to LSS10 included a FVIII activity peak sample (15–30 min) and a trough sample (166–168 h), with a third sample collected at one of the following time windows: 22–26, 46–50, 70–74, 94–98, 118–122 or 142–146 h.

A rich reference sampling strategy (RSS), with samples taken at 15–30 min, 46–50, 70–74, 94–98, 118–122, 142–146 and 166–168 h, was used as a benchmark to assess improvements in predictive performance with a more intensive sampling approach.

### 2.3 Monte Carlo Simulations and Bayesian Estimation

By means of a Monte Carlo simulation, individual PK parameters were randomly sampled from their respective distributions in the PopPK model. All individuals received a virtual dose of efanesoctocog alfa at 50 IU/kg once weekly, rounded to the nearest vial size, with infusion durations randomly assigned between 1 and 10 minutes. Using the assigned CL, V and dose, a steady‐state FVIII activity‐time curve was generated for each individual. Samples were randomly drawn from these curves within the intervals of the LSSs and RSS. Individual PK parameters were estimated by Bayesian estimation, applying the M3 method for samples below the assumed lower limit of quantification (BLOQ; <1 IU/dL). All simulations and estimations were performed using NONMEM v7.6.0.

### 2.4 Assessment of LSS Predictive Performance

The predictive performance of each LSS and the RSS was evaluated by assessing bias and precision in predicting individual PK parameters (CL, V, half‐life (t_½_)) and exposure metrics (FVIII activity peak level at 0.5 h (C_0.5_), C_168_ and the time FVIII activity remained above thresholds of 5, 10 and 40 IU/dL). Bias was calculated by the relative mean prediction error (rMPE) [Equation 1], precision was calculated by the relative root mean square error (rRMSE) [Equation 2]. A rMPE within −5% to 5% and rRMSE <25% were deemed adequate, consistent with other published factor concentrate LSS studies [13, 14, 15]. In addition, relative prediction errors were calculated for all PK parameters and exposure metrics [Equation 3].

(1) $$\mathrm{rMPE}\;\left(\%\right)=\frac{1}{n}\;\sum_{i=1}^{n}\frac{{\hat{\theta }}_{\mathrm{ij}}-\;\theta_{\mathrm{true},\;i}}{\theta_{\mathrm{true},\;i}}\times \;100\%\;$$

(2) $$\mathrm{rRMSE}\;\left(\%\right)=\sqrt{\frac{1}{n}\;\sum_{i=1}^{n}\;{\left(\frac{{\hat{\theta }}_{\mathrm{ij}}-\;\theta_{\mathrm{true},\;i}}{\theta_{\mathrm{true},\;i}}\right)}^{2}\times \;\;100\%}$$

(3) $$\mathrm{rela}\mathrm{tive}\;\mathrm{pred}\mathrm{icti}\mathrm{on}\;\mathrm{error}\;\left(\%\right)=\frac{{\hat{\theta }}_{\mathrm{ij}}-\;\theta_{\mathrm{true},\;i}}{\theta_{\mathrm{true},\;i}}\;\times \;100\%$$

In which n is the number of subjects, ${\hat{\theta }}_{ij}$ describes the estimated individual PK parameter or exposure metric for the i^th^ individual with the j^th^ LSS using Bayesian forecasting and $\theta_{true,\;i}$ is the true PK parameter or exposure metric obtained by the Monte Carlo simulation for the i^th^ individual.

In addition, for a LSS to be suitable for PK‐guided prophylactic dosing, at least 80% of patients needed an estimated C_168_ within ±3 IU/dL of the true C_168_ (prediction error). For PK‐guided pre‐operative dosing, at least 80% of patients required an estimated C_0.5_ within ±20 IU/dL of the true C_0.5_. These criteria ensure that the prediction error of C_168_ and C_0.5_ falls within predefined limits for at least 80% of patients, allowing the PK‐guided dosing strategy to be prospectively adjusted to reliably achieve target FVIII activity levels.

The time above a target level at steady‐state was calculated by rearranging the steady‐state plasma concentration equation after intravenous bolus administration [C_ss_, Equation (4)] to solve for t [Equation (5)].

(4) $$C_{ss}=\frac{D}{V}\;\times \;\frac{e^{-kt}}{1-e^{-k\tau }}$$

(5) $$t=-\frac{1}{k}\;\times \ln\left(\frac{C_{ss}\cdot V}{D}\times \left(1-e^{-k\tau }\right)\right)$$

In which D is the dose (IU), V is the volume of distribution (dL), k is the elimination rate constant (h^−1^), t is the time (h) and τ is the dosing interval (h). C_ss_ is the concentration at steady‐state (IU/dL) and was used to set a target FVIII activity level of 5, 10 and 40 IU/dL.

## 3 Results

### 3.1 Virtual Population

**FIGURE 1:**
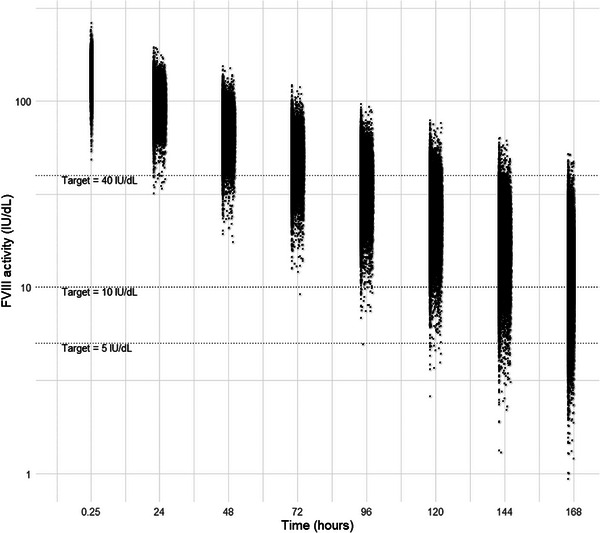
Simulated FVIII activity (IU/dL) over one dosing interval (*t* = 168 h) for the total virtual population. One simulated data point is shown per individual for the intervals 0.25–0.5, 22–26, 46–50, 70–74, 94–98, 118–122, 142–146 and 166–168 h. The three horizontal dotted lines indicate the FVIII activity target thresholds of 5, 10 and 40 IU/dL.

The virtual population closely resembled the population used by Wong et al. [10] for PopPK model development in terms of bodyweight (median [5^th^–95^th^ percentile]: 71.7 kg [25.2–93.4] vs. 72 kg [17.5–113.0]) and the proportion of Asians (10.6% vs. 15.4%). For each of these patients, a FVIII activity over time curve was constructed using Monte Carlo simulations. One FVIII activity level per patient per time interval (0.25–0.5, 22–26, 46–50, 70–74, 94–98, 118–122, 142–146 and 166–168 h) is shown in Figure 1. At 72 h, 75.4% of the virtual population was above the FVIII activity target threshold level of 40 IU/dL, while at 168 h, 64.5% and 94.5% of the population exceeded the target threshold levels of 10 and 5 IU/dL, respectively. Only 0.03% of the simulated FVIII activity levels were BLOQ.

### 3.2 Predictive Performance

All evaluated LSSs met the predefined criteria for bias (rMPE within ±5%) and precision (rRMSE <25%) for predicting CL, V, t_1/2_, C_168_ and C_0.5_ (Table 2). Consequently, bias and precision in the estimated time above FVIII activity target thresholds of 5, 10 and 40 IU/dL, which depend on CL and V, also met these criteria (Table 3).

**TABLE 2:** Bias (rMPE) including the 95% confidence interval and precision (rRMSE) for the predicted PK parameters CL, V and t_1/2_, and the exposure metrics C_168_ and C_0.5_ assessed for the RSS and LSSs for efanesoctocog alfa.

	Clearance (CL)		Volume of distribution (V)		Terminal half‐life (t_1/2_)		Predicted FVIII activity trough level at 168 h (C_168_)		Predicted FVIII activity peak level at 0.5 h (C_0.5_)	
Sampling strategy	rMPE (%) (95% CI)	rRMSE (%)	rMPE (%) (95% CI)	rRMSE (%)	rMPE (%) (95% CI)	rRMSE (%)	rMPE (%) (95% CI)	rRMSE (%)	rMPE (%) (95% CI)	rRMSE (%)
*7 samples*										
RSS	0.9 (0.8 to 1)	6.3	0.6 (0.4 to 0.8)	9.9	−0.4 (−0.5 to −0.3)	6.4	−0.7 (−0.9 to −0.5)	11.2	0.0 (−0.1 to 0.2)	8.5
*1 sample*										
LSS1	2.0 (1.8 to 2.2)	11.1	0.7 (0.5 to 0.9)	12.4	−1.1 (−1.2 to −0.9)	8.5	−1.2 (−1.5 to 0.8)	20.3	0.2 (0.0 to 0.4)	11.3
LSS2	2.6 (2.4 to 2.8)	11.3	0.7 (0.5 to 1.0)	12.4	−1.5 (−1.7 to −1.3)	10.0	−1.9 (−2.3 to 1.5)	22.3	−0.1 (−0.2 to 0.1)	10.9
*2 samples*										
LSS3	1.5 (1.3 to 1.6)	9.3	0.7 (0.5 to 0.9)	12.3	−0.8 (−0.9 to −0.7)	7.6	−1.2 (−1.4 to 0.9)	15.1	0.3 (0.1 to 0.5)	11.0
LSS4	2.1 (2.0 to 2.3)	10.2	0.8 (0.7 to 1.0)	10.9	−1.0 (−1.2 to −0.9)	8.4	−1.3 (−1.6 to 0.9)	20.4	−0.3 (−0.4 to −0.1)	9.7
*3 samples*										
LSS5	1.8 (1.7 to 2.0)	8.9	0.5 (0.3 to 0.7)	9.7	−1.0 (−1.2 to −0.9)	8.3	−1.2 (−1.5 to 0.8)	19.6	−0.1 (−0.3 to 0.0)	8.6
LSS6	1.7 (1.5 to 1.8)	8.7	0.5 (0.3 to 0.7)	10.0	−1.0 (−1.1 to −0.8)	8.1	−1.2 (−1.5 to 0.9)	18.4	−0.1 (−0.2 to 0.1)	8.8
LSS7	1.7 (1.5 to 1.9)	9.1	0.7 (0.5 to 0.9)	11.3	−0.9 (−1.1 to −0.8)	8.0	−1.3 (−1.6 to 1.0)	17.0	0.0 (−0.1 to 0.2)	10.0
LSS8	1.6 (1.5 to 1.7)	8.3	0.7 (0.5 to 0.9)	10.5	−0.8 (−1.0 to −0.7)	7.5	−1.3 (−1.5 to 1.0)	15.9	−0.1 (−0.3 to 0.1)	9.2
LSS9	1.5 (1.4 to 1.7)	8.3	0.7 (0.5 to 0.9)	10.6	−0.7 (−0.9 to −0.6)	7.3	−1.2 (−1.5 to 0.9)	15.1	−0.1 (−0.2 to 0.1)	9.3
LSS10	1.5 (1.4 to 1.7)	8.2	0.8 (0.6 to 1.0)	10.5	−0.7 (−0.8 to −0.6)	6.8	−1.2 (−1.5 to 1.0)	14.2	−0.2 (−0.3 to 0.0)	9.2

Abbreviations: CI, confidence interval; LSS, limited sampling strategy; rMPE, relative mean prediction error; rRMSE, relative root mean square error; RSS, reference sampling strategy.

**TABLE 3:** Bias (rMPE) including the 95% confidence interval and precision (rRMSE) for the predicted time that the FVIII activity was above a threshold of 5, 10 and 40 IU/dL assessed for the RSS and LSSs for efanesoctocog alfa.

	Time >5 IU/dL		Time >10 IU/dL		Time >40 IU/dL	
Sampling strategy	rMPE (%) (95% CI)	rRMSE (%)	rMPE (%) (95% CI)	rRMSE (%)	rMPE (%) (95% CI)	rRMSE (%)
*7 samples*						
RSS	−0.5 (−0.6 to −0.5)	4.9	−0.6 (−0.6 to −0.5)	4.7	−0.5 (−0.6 to −0.4)	5.6
*1 sample*						
LSS1	−1.2 (−1.3 to −1.1)	7.9	−1.2 (−1.3 to −1.1)	7.9	−0.8 (−1.0 to −0.6)	10.3
LSS2	−1.7 (−1.9 to −1.5)	9.0	−1.7 (−1.9 to −1.6)	9.1	−1.5 (−1.7 to −1.3)	10.7
*2 samples*						
LSS3	−0.9 (−1.0 to −0.8)	6.3	−0.9 (−1.0 to −0.8)	6.2	−0.5 (−0.7 to −0.4)	8.3
LSS4	−1.2 (−1.4 to −1.1)	7.9	−1.3 (−1.4 to −1.1)	7.9	−1.2 (−1.3 to −1.0)	9.7
*3 samples*						
LSS5	−1.2 (−1.3 to −1.1)	7.7	−1.2 (−1.3 to −1.1)	7.6	−1.1 (−1.3 to −1.0)	8.8
LSS6	−1.1 (−1.3 to −1.0)	7.4	−1.2 (−1.3 to −1.0)	7.3	−1.1 (−1.2 to −0.9)	8.4
LSS7	−1.1 (−1.2 to −1.0)	7.0	−1.1 (−1.2 to −1.0)	6.9	−0.9 (−1.1 to −0.8)	8.4
LSS8	−1.0 (−1.1 to −0.9)	6.6	−1.0 (−1.1 to −0.9)	6.5	−0.9 (−1.1 to −0.8)	7.8
LSS9	−0.9 (−1.0 to −0.8)	6.3	−0.9 (−1.1 to −0.8)	6.2	−0.8 (−0.9 to −0.7)	7.7
LSS10	−0.9 (−1.0 to −0.8)	5.8	−0.9 (−1.0 to −0.8)	5.8	−0.8 (−1.0 to −0.7)	7.4

Abbreviations: CI, confidence interval; LSS, limited sampling strategy; rMPE, relative mean prediction error; rRMSE, relative root mean square error; RSS, reference sampling strategy.

**FIGURE 2:**
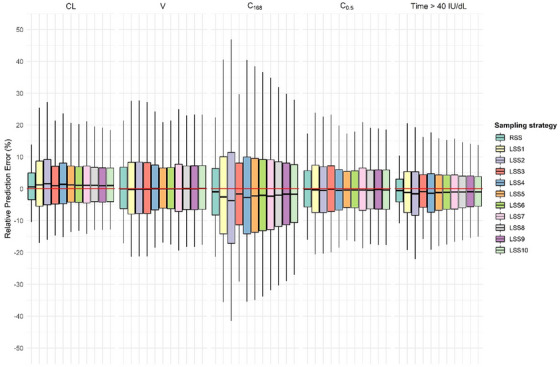
Distribution of the relative prediction errors of estimates from Bayesian analysis. The relative prediction error (%) is shown for the estimation of clearance (CL), volume of distribution (V), FVIII activity trough level at 168 h (C_168_), FVIII activity peak level at 0.5 h (C_0.5_) and the time spent above a FVIII activity target threshold of 40 IU/dL. In the boxplots, the median is denoted by the central black line within the box (IQR, 25^th^–75^th^ percentiles), and the whiskers represent the 5^th^ and 95^th^ percentiles. The red line at 0% represents the line of no systematic bias, indicating perfect agreement of the estimates with the simulated values on average.

However, all LSSs slightly overestimated CL (indicated by a positive rMPE; Table 2, and a positive median relative prediction error; Figure 2) while underestimating t_1/2_, C_168_ and time above the FVIII activity thresholds (indicated by a negative rMPE; Tables 2 and 3, and a negative median relative prediction error; Figure 2). In contrast, V and C_0.5_ estimates were nearly unbiased across all LSSs, with the rMPEs and median relative prediction errors close to zero (Table 2 and Figure 2).

The relative prediction errors were similar across the LSSs for CL, V, C_0.5_ and time above a FVIII activity threshold of 40 IU/dL (Figure 2). For C_168_, the 5^th^–95^th^ percentile range appeared narrower across the LSSs, from –41.5% to 46.9% in the widest LSS2 to –27.1% to 27.9% in the most optimal LSS10; however, this primarily reflects improvements in predicting low FVIII activity levels, where small absolute differences resulted in relatively large reductions in relative prediction error.

**FIGURE 3:**
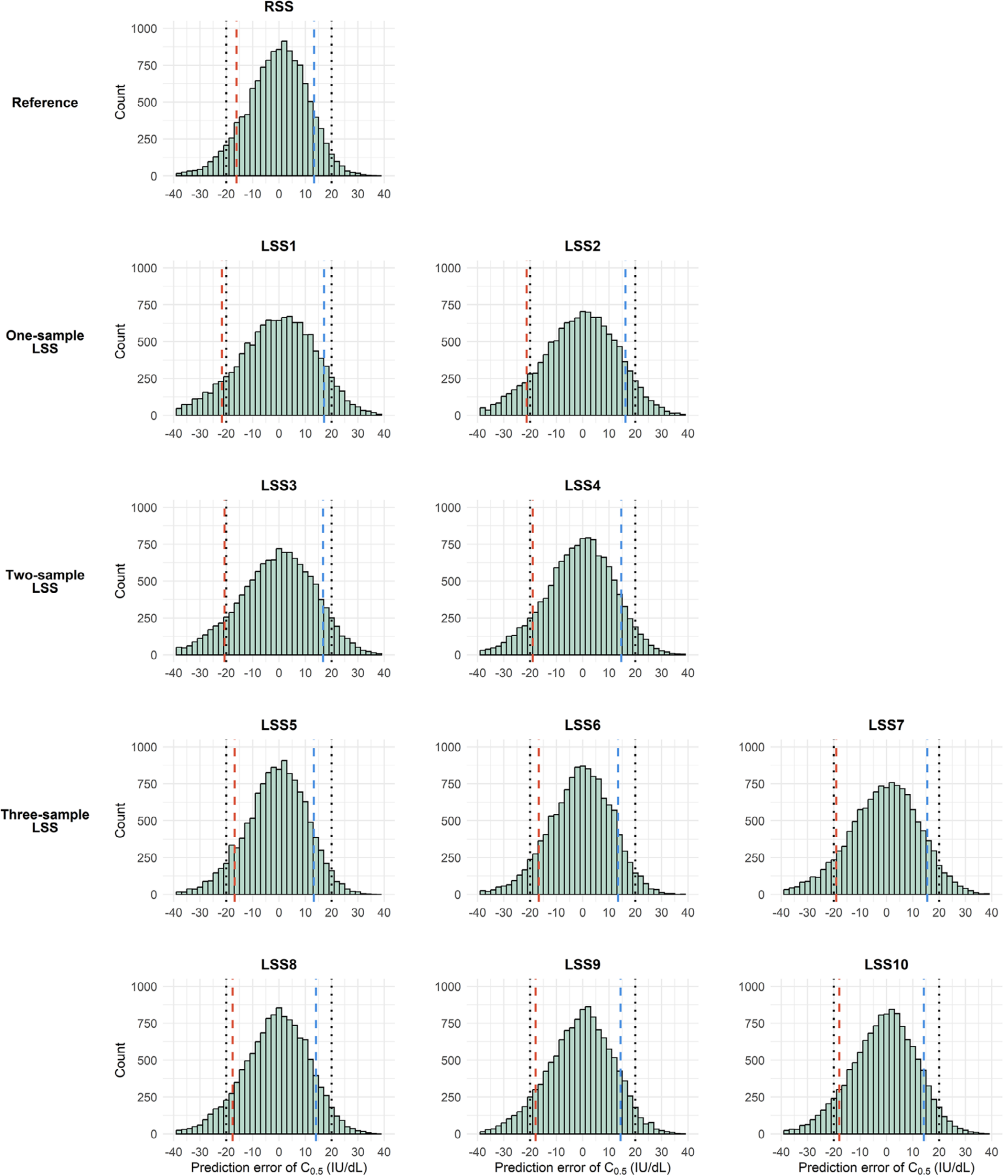
Frequency distribution of the prediction error of C_0.5_ shown for the RSS, and one‐, two‐ and three‐sample LSSs. The vertical dashed red lines indicate the 10^th^ percentile (Q10) and dashed blue lines indicate the 90^th^ percentile (Q90) of the prediction error distribution for each strategy. The black dotted lines mark the tolerated ±20 IU/dL prediction error window for the LSS to be suitable for pre‐operative PK‐guided dosing advice.

**FIGURE 4:**
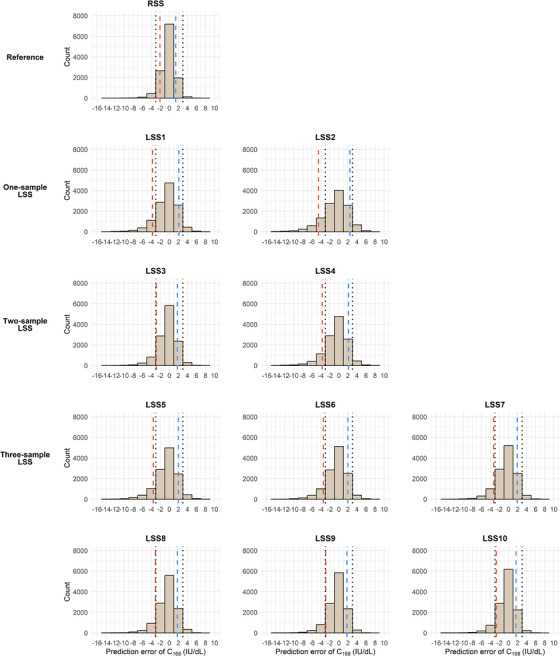
Frequency distribution of the prediction error of C_168_ shown for the RSS, and one‐, two‐ and three‐sample LSSs. The vertical dashed red lines indicate the 10^th^ percentile (Q10) and dashed blue lines indicate the 90^th^ percentile (Q90) of the prediction error distribution for each strategy. The black dotted lines mark the tolerated ±3 IU/dL prediction error window for the LSS to be suitable for prophylactic PK‐guided dosing advice.

The distributions of the prediction error of C_0.5_ and C_168_ across the RSS and LSSs are shown in Figures 3 and 4. For C_0.5_, LSS4–LSS10 met the requirement of 80% of the estimated values within ±20 IU/dL of the true values (10^th^–90^th^ percentile range; Figure 3). For C_168_, LSS3, LSS8, LSS9 and LSS10 showed at least 80% of the estimated values within ±3 IU/dL of the true value (10^th^–90^th^ percentile range; Figure 4). Importantly, adequate predictive performance across the entire C_168_ range does not necessarily guarantee that prediction errors will stay within ±3 IU/dL for low C_168_. As PK‐guided dosing targets low FVIII levels and/or longer intervals, the subset of patients with C_168_ < 5 IU/dL (*n* = 691) becomes particularly relevant. For this subset, the mean (SD) prediction errors were 0.38 (0.76) IU/dL in LSS3 and 0.36–0.46 (0.71–0.83) IU/dL in LSS8–10.

The seven‐sample RSS, which is an invasive sampling strategy requiring six hospital visits, showed only relatively small improvements in bias, precision and (relative) prediction errors compared with the one‐, two‐ and three‐sample LSSs (Tables 2 and 3 and Figures 3 and 4).

## 4 Discussion

In this study, a RSS and 10 LSSs were evaluated using simulations of 12,500 virtual haemophilia A patients’ FVIII activity–time profiles for efanesoctocog alfa administered at the prophylactic regimen of 50 IU/kg once weekly. We assessed which LSSs were suitable to provide PK‐guided prophylactic and pre‐operative dosing advice in patients already receiving this prophylactic regimen. For PK‐guided prophylactic dosing advice, LSS3 (samples at 118–122 h and 166–168 h), LSS8 (samples at 0.25–0.5 h, 94–98 h and 166–168 h), LSS9 (samples at 0.25–0.5 h, 118–122 h and 166–168 h) and LSS10 (samples at 0.25–0.5 h, 142–146 h and 166–168 h) were deemed suitable. For pre‐operative dosing advice, LSS4 (samples at 0.25–0.5 h and 166–168 h) and LSS5–10 were considered suitable. Each of LSS5‐10 included samples at 0.25–0.5 h and 166–168 h, plus one additional sample taken at either 22–26, 46–50, 70–74, 94–98, 118–122, or 142–146 h. The minor improvements in the predictive performance of the RSS compared to the LSSs demonstrate that a more invasive 7‐sample sampling strategy is not necessary for accurate and precise PK‐guided dosing.

Since several LSSs performed adequately, clinicians have the flexibility to choose the strategy that best suits the treatment setting, patients’ preferences and potential challenges with venous access. Given the minimal dosing frequency required with efanesoctocog alfa, extensive intravenous blood sampling is often less acceptable to patients.

For prophylaxis, PK‐guided dosing for efanesoctocog alfa should aim to achieve a clinician‐defined FVIII activity trough level and ensure sufficient time above a specific target to prevent spontaneous and activity‐induced bleeding. These targets should be individualised based on the patient's lifestyle, physical activity, bleeding phenotype and the estimated FVIII activity levels observed in previous bleeding episodes. An initial PK profile can be used to tailor the prophylactic dosing regimen to the defined targets. For this, a three‐sample LSS (LSS8‐10) is recommended, followed by target verification using the two‐sample LSS3.

To determine the pre‐operative dose needed, LSS4‐10 are recommended. Post‐operatively, monitoring and adjustments should be guided by clinical protocols, considering the type of surgery and the patient's recovery. Clinicians should be aware that it is currently unclear whether the PK of efanesoctocog alfa differs perioperatively, as changes in PK have been observed with other FVIII concentrates, which could impact the optimal LSS and treatment [16].

For dose determination of efanesoctocog alfa in a bleeding setting, we recommend applying the LSSs identified as suitable for pre‐operative use only in cases of light bleeding, when the physician's goal is to achieve a defined peak level. For more severe bleeding events, the WFH recommends maintaining a sustained FVIII activity level over time [4]. However, we have not evaluated the bias, precision and (relative) prediction error of the LSSs across FVIII activity levels other than C_0.5_ and C_168_, and therefore cannot provide recommendations for their use in these scenarios.

The requirements for the prediction error can also be applied as a practical strategy for PK‐guided dosing. By requiring that the C_168_ predictions of 80% of patients fall within a prediction error of ±3 IU/dL, clinicians can target a C_168_ of 6 IU/dL for prophylactic dosing, resulting in a C_168_ between 3 and 9 IU/dL for 80% of patients. Such an approach broadly aligns with the WFH‐recommended trough range of 3–5 IU/dL [4]. In the pre‐operative setting, the same principle applies: if a clinician sets a pre‐operative target of 80–100 IU/dL, aiming for the upper end of 100 IU/dL ensures that 80% of patients will reach a C_0.5_ within 80–120 IU/dL using the LSSs that were deemed adequate in our study.

These recommendations are derived from this LSS in silico study and should be viewed as guidance rather than direct clinical instructions. Differences in patient populations and real‐world conditions may alter predictions, and prospective clinical validation of both the PopPK model and the LSSs is warranted.

In the future, PopPK models for efanesoctocog alfa that link the PK to the pharmacodynamics should be further explored as PK‐guided dosing assumes a direct correlation between factor levels and haemostatic control, which does not always apply, as demonstrated for other FVIII concentrates [17, 18, 19].

## 5 Conclusion

In conclusion, several developed LSSs can support PK‐guided dosing of efanesoctocog alfa in clinical practice, potentially enabling more informed treatment decisions in the prophylactic, pre‐operative and some on‐demand settings.

## Author Contributions

Jelien den Hollander: contributed to the design, analysis and writing of the research, performed the research. Marjon H. Cnossen: contributed to the design, analysis and writing of the research. Ron A. A. Mathôt: contributed to the design, analysis and writing of the research.

## Funding

This study was performed on behalf of the SYMPHONY consortium (NWA.1160.18.038.), which is funded by a grant from the Netherlands Organisation for Scientific Research (NWO) and is supported by sponsorship from Swedish Orphan Biovitrum, CSL Behring and Bayer.

## Ethics Statement

The authors have nothing to report.

## Conflicts of Interest

J.H. has no interests which might be perceived as posing a conflict or bias. M.H.C. has received investigator‐initiated research and travel grants as well as speaker fees over the years from the Netherlands Organisation for Scientific Research (NWO) and Netherlands National research Agenda (NWA), the Netherlands Organization for Health Research and Development (ZonMw), the Dutch Innovatiefonds Zorgverzekeraars, Stichting Haemophilia, Baxter/Baxalta/Shire/Takeda, Pfizer, Bayer Schering Pharma, CSL Behring, Sobi, Biogen, Novo Nordisk, Novartis, Roche and Nordic Pharma, and for serving as a steering board member for Roche, Bayer and Novartis. All grants, awards and fees go to Erasmus MC as an institution. She is the coordinator of Erasmus MC as a Health Care Provider within the European Reference Network (ERN) for rare haematological diseases EuroBloodNet, and (co)leader of the local Erasmus MC Expert Centres for Rare Bleeding Disorders and Sickle Cell and Thalassemia Comprehensive Care Centre. R.A.A.M. has received grants from governmental and societal research institutes such as NWO, ZonMW, Dutch Kidney Foundation and Innovation Fund and unrestricted investigator research grants from Baxter/Baxalta/Shire/Takeda, Bayer, CSL Behring, Octapharma, Sobi and CelltrionHC. He has served as advisor for Bayer, CSL Behring, Octapharma, Sobi and CelltrionHC. All grants and fees are paid to the Amsterdam UMC as an institution.

## Data Availability

The data that support the findings of this study are available from the corresponding author upon reasonable request. Data remains available in the Amsterdam University Medical Centres and is guarded for access on demand for 15 years according to national regulations.
